# Mechanical power is associated with cardiac output and pulmonary blood flow in an experimental acute respiratory distress syndrome in pigs

**DOI:** 10.3389/fphys.2024.1462954

**Published:** 2024-10-15

**Authors:** Yingying Zhang, Jakob Wittenstein, Anja Braune, Raphael Theilen, Lorenzo Maiello, Giulia Benzi, Thomas Bluth, Thomas Kiss, Xi Ran, Thea Koch, Patricia R. M. Rocco, Marcus J. Schultz, Jörg Kotzerke, Marcelo Gama De Abreu, Robert Huhle, Martin Scharffenberg

**Affiliations:** ^1^ Department of Anesthesiology and Intensive Care Medicine, Pulmonary Engineering Group, Faculty of Medicine and University Hospital Carl Gustav Carus, TUD Dresden University of Technology, Dresden, Germany; ^2^ Department of Anesthesiology, Affiliated Hospital of Southwest Medical University, Luzhou, China; ^3^ Department of Nuclear Medicine, Faculty of Medicine and University Hospital Carl Gustav Carus, TUD Dresden University of Technology, Dresden, Germany; ^4^ Anesthesia and Critical Care, San Martino Policlinico Hospital, IRCCS for Oncology and Neurosciences, Genoa, Italy; ^5^ Department of Clinical and Biological Sciences “Ospedale di Circolo e Fondazione Macchi”, Service of Anesthesia and Intensive Care, University of Insubria, Varese, Italy; ^6^ Department of Anesthesiology, Intensive-, Pain- and Palliative Care Medicine, Radebeul Hospital, Academic Hospital of the Technische Universität Dresden, Radebeul, Germany; ^7^ Department of Intensive Care, Chongqing General Hospital, University of Chinese Academy of Science, Chongqing, China; ^8^ Laboratory of Pulmonary Investigation, Carlos Chagas Filho Institute of Biophysics, Federal University of Rio de Janeiro, Rio deJaneiro, Brazil; ^9^ Department of Intensive Care and Laboratory of Experimental Intensive Care and Anesthesiology, Academic Medical Center, University of Amsterdam, Amsterdam, Netherlands; ^10^ Department of Intensive Care and Resuscitation, Cleveland Clinic, Anesthesiology Institute, Cleveland, OH, United States; ^11^ Department of Outcomes Research, Cleveland Clinic, Anesthesiology Institute, Cleveland, OH, United States

**Keywords:** ARDS, cardiac output, mechanical power, pulmonary blood flow, ventilator-induced lung injury, ^68^Gallium, jacobian determinant

## Abstract

**Background:**

Despite being essential in patients with acute respiratory distress syndrome (ARDS), mechanical ventilation (MV) may cause lung injury and hemodynamic instability. Mechanical power (MP) may describe the net injurious effects of MV, but whether it reflects the hemodynamic effects of MV is currently unclear. We hypothesized that MP is also associated with cardiac output (CO) and pulmonary blood flow (PBF).

**Methods:**

24 anesthetized pigs with experimental acute lung injury were ventilated for 18 h according to one of three strategies: 1) Open lung approach (OLA), 2) ARDS Network high-PEEP/F_I_O_2_ strategy (HighPEEP), or 3) low-PEEP/F_I_O_2_ strategy (LowPEEP). Total MP was assessed as the sum of energy dissipated to overcome airway resistance and energy temporarily stored in the elastic lung tissue per minute. The distribution of pulmonary perfusion was determined by positron emission tomography. Regional PBF and MP, assessed in three iso-gravitational regions of interest (ROI) with equal lung mass (ventral, middle, and dorsal ROI), were compared between groups.

**Results:**

MP was higher in the LowPEEP than in the OLA group, while CO did not differ between groups. After 18 h, regional PBF did not differ between groups. During LowPEEP, regional MP was higher in the ventral ROI compared to OLA and HighPEEP groups (2.5 ± 0.3 vs. 1.4 ± 0.4 and 1.6 ± 0.3 J/min, respectively, P < 0.001 each), and higher in the middle ROI compared to the OLA group (2.5 ± 0.4 vs. 1.6 ± 0.5 J/min, *P* = 0.04). MP in the dorsal ROI did not differ between groups (1.4 ± 0.9 vs. 1.4 ± 0.5 vs. 1.3 ± 0.8 J/min, *P* = 0.916). Total MP was independently associated with CO [0.34 (0.09, 0.59), *P* = 0.020]. Regional MP was positively associated with PBF irrespective of the regions [0.52 (0.14, 0.76), *P* = 0.01; 0.49 (0.10, 0.74), *P* = 0.016; 0.64 (0.32, 0.83), *P* = 0.001 for ventral, middle, and dorsal ROI, respectively]. Subgroup analysis revealed a significant association of MP and CO only in the OLA group as well as a significant association between MP with regional PBF only in the HighPEEP group.

**Conclusion:**

In this model of acute lung injury in pigs ventilated with either open lung approach, high, or low PEEP tables recommended by the ARDS network, MP correlated positively with CO and regional PBF, whereby these clinically relevant lung-protective ventilation strategies influenced the associations.

## Introduction

Mechanical ventilation (MV) is a life-saving therapy and is frequently used in intensive care units, particularly for patients with Acute Respiratory Distress Syndrome (ARDS). However, the increased intrathoracic pressure (ITP) may impair cardiovascular function and cause life-threatening hemodynamic instability, while the excessive strain and stress may contribute to ventilator-induced lung injury (VILI), further compromising gas exchange. Lung-protective ventilation strategies limiting distending pressures and volumes, and reduce repetitive alveolar opening and closing, have been well studied, but less is known about the impact of hemodynamically relevant changes on the development of VILI through their effects on pulmonary circulation (Slutsky and Ranieri, 2013).

Previous studies have found that damages secondary to either high vascular or alveolar pressure were less pronounced in the absence of tidal positive ventilation, suggesting that periodic elevation of pulmonary artery pressure can further impair the alveolar-capillary membrane permeability (Hotchkiss et al., 2001; Katira, 2019).

Traction forces applied to the junction tissue, which forms the interface between collapsed and ventilated alveoli, can amplify the stress caused by capillary compression, leading to increased transmural pressure that may result in stress fractures (Hotchkiss et al., 2001; Marini et al., 2003; Marini et al., 2024). Consequently, the deformed endothelial cells and the augmented cytoskeletal tension, as well as the amplified shearing stress may promote edema formation, perivascular hemorrhage, alveolar hemorrhage, and albumin leakage, further worsening lung damage (Hotchkiss et al., 2000; Lopez-Aguilar et al., 2006). Moreover, higher pulmonary vascular flow in isolated perfused rabbit lungs, along with increased cardiac output (CO) induced by dopamine administration, may contribute to disproportionate vascular injury beyond the scope of VILI (Dreyfuss and Saumon, 1993; Dreyfuss et al., 1988; Lopez-Aguilar et al., 2006).

Given that CO modulates the magnitude of pulmonary blood flow (PBF), and that passive dilation and closure of pulmonary capillaries cause fluctuations in pulmonary artery pressure, affecting PBF, it would be valuable to identify a unifying variable to describe the overall effects of mechanical ventilation on PBF. Mechanical power (MP) is defined as the energy delivered from the ventilator to the respiratory system per minute (Gattinoni et al., 2016) and has been identified as an independent risk factor both for VILI and mortality in experimental (Collino et al., 2019; Moraes et al., 2018; Scharffenberg et al., 2021) and clinical ARDS (Chi et al., 2021; Costa et al., 2021; Cressoni et al., 2016; Xie et al., 2022), respectively. However, the effects of MP on CO and PBF have not been widely studied. Therefore, we aimed to determine the effects of different strategies on respiratory and hemodynamic variables, as well as the distribution of ventilation and perfusion in pigs with experimental ARDS, and investigate the effects of MP on CO and regional PBF. We hypothesized that MP is associated with CO and regional PBF, and that different ventilation strategies modulate these associations.

## Materials and methods

### Protocol approval

The Institutional Animal Care and Welfare Committee and the Government of the State of Saxony, Germany (25-5131/474/31; 27.09.2019) approved the study. All animals received humane care in compliance with the Principles of Laboratory Animal Care formulated by the National Society for Medical Research and the US National Academy of Sciences Guide for the Care and Use of Laboratory Animals and complied with relevant aspects of the ARRIVE guidelines.

### Animal preparation and mechanical ventilation

Twenty-four juvenile female pigs (German landrace, Danish Specific Pathogen Free Certification, www.spf.dk) were intramuscularly sedated with Midazolam (1 mg kg^−1^) and Ketamine (15 mg kg^−1^), oro-tracheally intubated, and mechanically ventilated under volume-controlled mode (Evita XL, Dräger, Germany). The following initial settings were used: Tidal volume (V_T_) 6 mL kg^−1^, PEEP 5 cmH_2_O, inspiratory: expiratory (I: E) ratio 1:1, fraction of inspired oxygen (F_I_O_2_) 1.0, flow 35 L/min, and respiratory rate (RR) adjusted to arterial pH within 7.35–7.45. V_T_ was reduced if airway plateau pressure (P_plat_) was ≥30 cmH_2_O. Total intravenous anesthesia using Ketamine (15 mg kg^−1^ h^−1^), Midazolam (1 mg kg^−1^ h^−1^), and Atracurium (3 mg kg^−1^ h^−1^) was adopted throughout the whole experiment. Crystalloids were infused at 10 mg kg^−1^ h^−1^ and 4 mg kg^−1^ h^−1^ during preparations and intervention time, respectively. Severe hemodynamic depression (mean arterial pressure, MAP, <60 mmHg) was primarily treated using Norepinephrine as well as using bolus infusion of crystalloids and colloids, as appropriately.

The right common carotid artery, external jugular vein, and urinary bladder were catheterized under sterile conditions with additional local anesthesia. A Swan-Ganz pulmonary artery catheter was placed as described elsewhere (Scharffenberg et al., 2021).

### Lung injury

Lung injury was induced by repetitive lung lavages with warmed 0.9% saline in prone and supine positions (37°C; 35 mL kg^−1^; lavage pressure approx. 30 cmH_2_O), until moderate ARDS criteria were achieved (PaO_2_/F_I_O_2_ < 200 mmHg maintained for at least 30 min), as described in detail elsewhere (Scharffenberg et al., 2021).

### Experimental protocol

**FIGURE 1:**
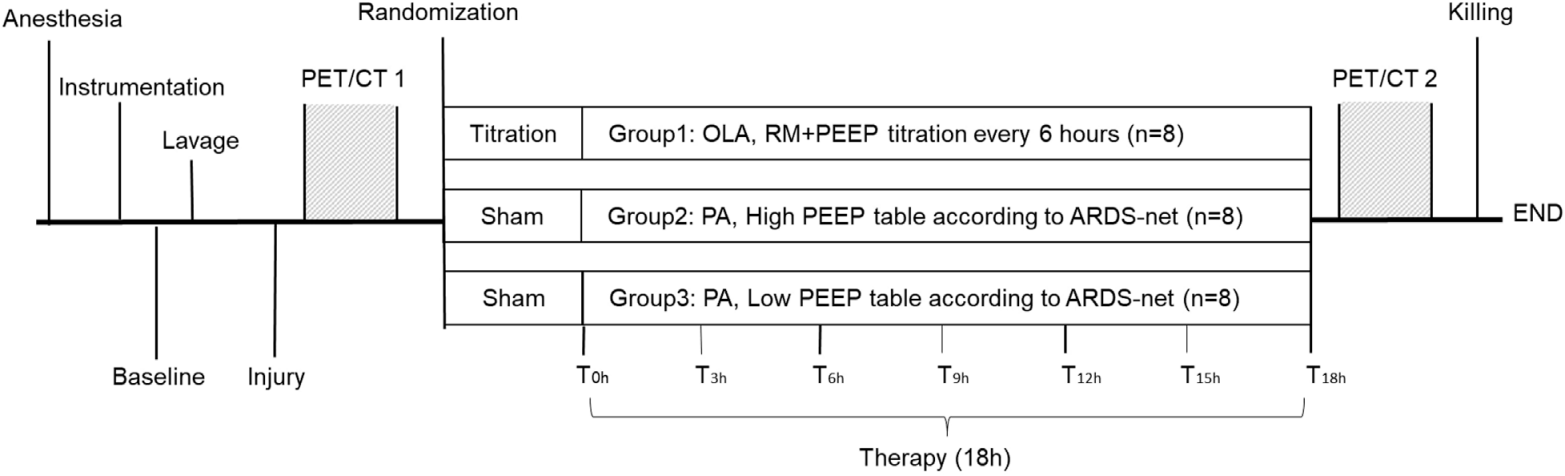
Schematic description of the experimental protocol. PA, permissive atelectasis; OLA, open lung approach; RM, recruitment maneuver; PEEP, positive end-expiratory pressure; T_0h_, the start of intervention time; T_3h–18h_, measurement time points every 3 h from T_0h_ onwards. 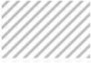: Lung imaging assessment at the Department of Nuclear Medicine.

The time course of the experiments is presented in Figure 1. After inducing lung injury (prior to randomization, PET/CT 1), and at the end of the intervention time (PET/CT 2), lung imaging was conducted at the Department of Nuclear Medicine (University Hospital Carl Gustav Carus, Dresden, Germany) using a digital Biograph Vision 600 PET/CT (Siemens Healthineers AG, Erlangen, Germany).

Following low dose CT scans used for attenuation correction of the following static PET scan (attenuation correction CT, ACCT, 120 kV, 35 mA, 5.88 s scan time, spiral pitch factor of 1.5, 440 × 440 matrix with a voxel size of (1.65 × 1.65 × 2) mm^3^, reconstruction kernel H31f), ^68^Gallium (^68^Ga)- labeled albumin microspheres were injected intraveneously and their distribution was assessed by a static ^68^Ga-PET scan for assessment of lung perfusion (continuous bed motion with a speed of 1.4 mm/s). Static whole lung spiral CTs (120 kV, eff. 120 mAs, spiral pitch factor of 1.35, 512 × 512 matrix with a voxel size of (0.98 × 0.98 × 1) mm^3^, reconstruction kernel B30f) at PEEP, mean airway pressure (P_mean_), and P_plat_ were sequentially conducted for the assessment of the distribution of lung aeration. Animals were positioned supine for all scans.

Animals assigned to OLA received recruitment maneuvers (RMs) and decremental PEEP titration to fully open the lung and achieve the highest compliance, respectively. This procedure was repeated every 6 h over the intervention time. Animals assigned to LowPEEP or HighPEEP were ventilated according to a fixed combination of PEEP/F_I_O_2_ that was recommended by the ARDS network to keep PaO_2_ of 55–80 mmHg (see methods, supplemental digital content, describing further details). RMs were not allowed for these two groups but ventilator settings were adjusted hourly according to the blood gas analysis (BGA). All animals were killed by intravenous bolus injection of 2 g thiopental and 50 mL 1 M potassium chloride after the second lung imaging.

### Measurements

Hemodynamic and gas exchange variables as well as respiratory signals were obtained prior to induction of lung injury (baseline), after establishing moderate ARDS (Injury), at the start of the intervention (T_0h_), and every 3 h after that until the last time point (T_0h_ to T_18h_). BGA were performed using a commercially available decive (ABL80, Radiometer, Denmark). Pulmonary hemodynamics were measured via the Swan-Ganz catheter. CO was assessed using the thermodilution method (MP70, Philips Healthcare, Netherlands), averaging three measurements spread over the respiratory cycle. Stroke volume (SV) and pulmonary vascular resistance (PVR) were calculated according to standard equations.

Airflow and airway pressure signals were recorded from the ventilator using a serial interface. Driving pressure (ΔP) was calculated as P_plat_ minus PEEP. Respiratory system elastance (E_rs_) and airway resistance (R_aw_) were derived by fitting the linear equation of motion to the acquired respiratory signals by multiple linear regression. MP was defined as the sum of energy dissipated to overcome airway resistance and energy temporarily stored in the elastic lung tissue in 1 min, and can be calculated with reference to the following equation (Huhle et al., 2019; Huhle et al., 2018; Scharffenberg et al., 2021).

$$MP=0.098•RR•V_{T}^{2}•\left[\frac{1}{2}•E_{rs}+RR•\frac{\left(1+I:E\right)}{\left(60•I:E\right)}•R_{aw}\right]$$ (1)

### Definition of regions of interest

Assuming that tissue density is linearly correlated with the number of blood vessels supplying the tissue (Arai et al., 2016) and to ensure that the number of blood vessels was similar between ROIs, we defined ROIs as three iso-gravitational planes with equivalent lung density along the ventrodorsal direction based on CT scans and named them ventral, middle, and dorsal regions.

### Aeration

Lung segmentation using a semi-automatic approach was conducted prior to analyzing the aeration data (Braune, 2017). A deep convolutional neural network algorithm and manual refinement were used for image co-registration to minimize image distortion and displacement, as described in detail elsewhere (Tustison and Avants, 2013). Lung volume was computed by multiplying the number of pixels determining the lungs by the pixel size and the slice thickness. The volumetric deformation at voxel i was quantified by Jacobian determinant (directional derivative) matrix J, which provides the ratio of the final to the initial volume of the co-registration and the co-registered end-expiratory (Motta-Ribeiro et al., 2019). The tidal volume at voxel i ( $V_{T,i}$ ) was then calculated according to Equation 2:

$$V_{T,i}=V_{i,endinsp}-V_{i,endexp}$$ (2)

Assuming constant homogenous end-inspiratory alveolar pressure and assuming no intrinsic PEEP, MP at voxel i ( ${MP}_{i,CT}$ ) at ^68^Ga-PET/CT resolution was calculated according to Equation 3:

$${MP}_{i,CT}=\frac{1}{2}•RR•V_{T,i}•\Delta P$$ (3)

Regional MP was equal to the sum of ${MP}_{i,CT}$ in the corresponding ROI.

### Regional PBF

PET data were reconstructed using an ordered subset expectation maximization (OSEM) 3D iterative reconstruction algorithm with six iterations and 5 subsets (6i5s) including point spread function modeling and time of flight measurements, with an image matrix size of 440 × 440, resulting in a voxel size of (1.65 × 1.65×2) mm^3^. No postfiltering was applied (all-pass filter). Reconstructions were performed with attenuation and absolute scatter correction. ^68^Ga net activity (^68^Ga-PET_net_) of the attenuation-corrected images was calculated by substraction of the background [^18^F]FDG activity, taking into account the decay half-lives of ^18^F and ^68^Ga. The transformation matrix obtained from deformable image registration was used to transform static CT and ^68^Ga-PET_net_ scans, and then the transformed CT scans with defined ROIs were co-registered to the transformed static ^68^Ga-PET_net_ scans to compute regional PBF (Klein et al., 2010; Shamonin et al., 2013).

Given that the total amount of PBF equals to CO, CO_Ga_ measured before ^68^Ga-PET scan was used to calculate the magnitude of PBF at the regional level. Thus, the absolute value of regional PBF (Q_abs, ROI_) was calculated according to Equation 4:

$$Q_{\text{abs},ROI}=\frac{68_{Ga-{PET}_{ROI}}}{68_{Ga}-{PET}_{TOTAL}}•{CO}_{Ga}$$ (4)

where ^68^Ga-PET_ROI_ represents the total ^68^Ga-PET_net_ inside the ROI, ^68^Ga-PET_TOTAL_ represents the total ^68^Ga-PET_net_ activity within the whole lung.

Considering that the induction of ARDS by repeated lavages can partly retain fluid in the airways and alveoli, thus leading to an overestimation of lung density at PET/CT-1 and, accordingly, an underestimation of regional PBF, while positive pressure ventilation and capillary/lymphatic resorption can remove lavage fluid over time (PET/CT-2), in order to make regional PBF measured at PET/CT-1 and PET/CT-2 comparable, we further normalized regional PBF with CT-derived lung mass according to Equation 5:

$$Q_{\text{abs},\text{nor}}=\frac{Q_{\text{abs},ROI}}{{lung\;mass}_{ROI}}$$ (5)

### Statistical analysis

This was a pre-planned sub-protocol of a larger experimental trial on the effects and mechanisms of MP and VILI as represented by PET/CT-based pulmonary neutrophilic inflammation. The sample size was based on the main protocol. Hemodynamics, respiratory variables, and gas exchange were analyzed using a general linear model (GLM) and a two-factorial ANOVA corrected for repeated measures with factors group and time. Pairwise *post hoc* multiple comparisons were performed according to Šidák. General aspects and lung imaging data were carried out by ANOVA for parametric data and Kruskal-Wallis for non-parametric data as appropriate with *P*-value adjusted to Šidák and Bonferroni. The mean values of MP and CO over the intervention were used for correlation analyses. Linear univariate regression was performed to determine the variables related to CO. The independent variables of interest (assumed *P* < 0.2) were diagnosed for covariance before multivariate regression analysis. The relevant ventilator variable was then entered into a linear multivariate regression model and adjusted. Pearson’s linear correlation analysis was used for the associations of regional MP with PBF. The discrimination of each model was expressed as correlation coefficients, reported as 95% confidence intervals [*r* (95% CI)]. Significance was accepted at a two-tailed *P*-value of <0.05. The statistical analysis was conducted with SPSS (Version 26, IBM Corp, United States). Graphs were prepared with GraphPad Prism (Version 8.0; GraphPad Software, San Diego, CA, United States).

## Results

Characteristics of animals, duration of anesthesia and mechanical ventilation as well as i. v. fluid and vasopressor doses are presented in the supplemental digital content.

### Respiratory variables and lung mechanics

**FIGURE 2:**
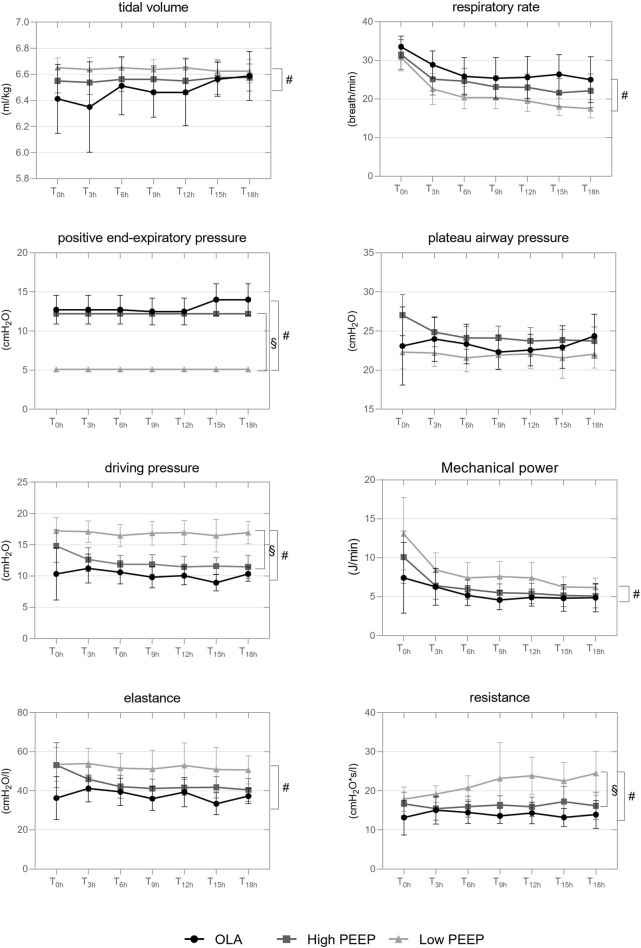
Time course of respiratory variables and lung mechanics throughout the intervention Interleaved symbols are presented in mean and SD. T_0h_, the start time of intervention; T_3h–18h_ are repeated measurements performed every 3 h from T_0h_ onwards. GLM was adopted for group comparison over intervention time (T_0h_-_18h_). #, comparison between OLA and LowPEEP; §, comparison between LowPEEP and HighPEEP groups. The correction according to Šidák was used.

Figure 2 depicts the time course of respiratory variables and mechanics. During the intervention time, animals ventilated with the LowPEEP strategy received slightly greater actual V_T_ and lower RR than the OLA group. As compared to both OLA and HighPEEP groups, PEEP (*P <* 0.001 for both) was lower and △P (*P <* 0.001 for both) and R_aw_ (*P <* 0.001 and *P =*0.004, respectively) were higher in the LowPEEP group. E_rs_ and MP were higher in the LowPEEP group as compared to the OLA group (*P* = 0.023 and *P* = 0.001, respectively). P_plat_ did not differ between groups, while mean airway pressure was lower during LowPEEP than both HighPEEP and OLA (see Supplementary Table S2, supplemental digital content).

At the end of intervention time, during LowPEEP, regional MP was higher in the ventral ROI as compared to OLA and HighPEEP groups (2.5 ± 0.3 vs. 1.4 ± 0.4 vs. 1.6 ± 0.3 J/min, respectively, *P <* 0.001 each) as well as higher in the middle ROI as compared to OLA group (2.5 ± 0.4 vs. 1.6 ± 0.5 J/min, *P =*0.04). MP distributed to the dorsal ROI did not differ between groups (1.4 ± 0.9 vs. 1.4 ± 0.5 vs. 1.3 ± 0.8 J/min, *P =*0.916). Gas exchange variables are presented elsewhere (see Supplementary Table S2, supplemental digital content).

### Hemodynamics

Except for SV, hemodynamic variables did not differ between groups over the intervention time (see Supplementary Table S3, supplemental digital content, summarizing hemodynamics). SV was greater in the LowPEEP group than in OLA and HighPEEP groups (*P =*0.002 and *P =*0.018, respectively). While CO_Ga_ did not differ before and at the end of intervention time, it significantly increased from 5.5 ± 1.0 to 8.6 ± 1.7 L/min in LowPEEP group (*P =*0.001) and from 5.1 ± 1.4 to 7.4 ± 2.1 L/min in HighPEEP group (*P =*0.004). CO_Ga_ in the OLA group did not differ between the two measurements (5.6 ± 1.8 vs. 6.2 ± 2.1 L/min, *P =*0.546).

### Regional pulmonary perfusion

**FIGURE 3:**
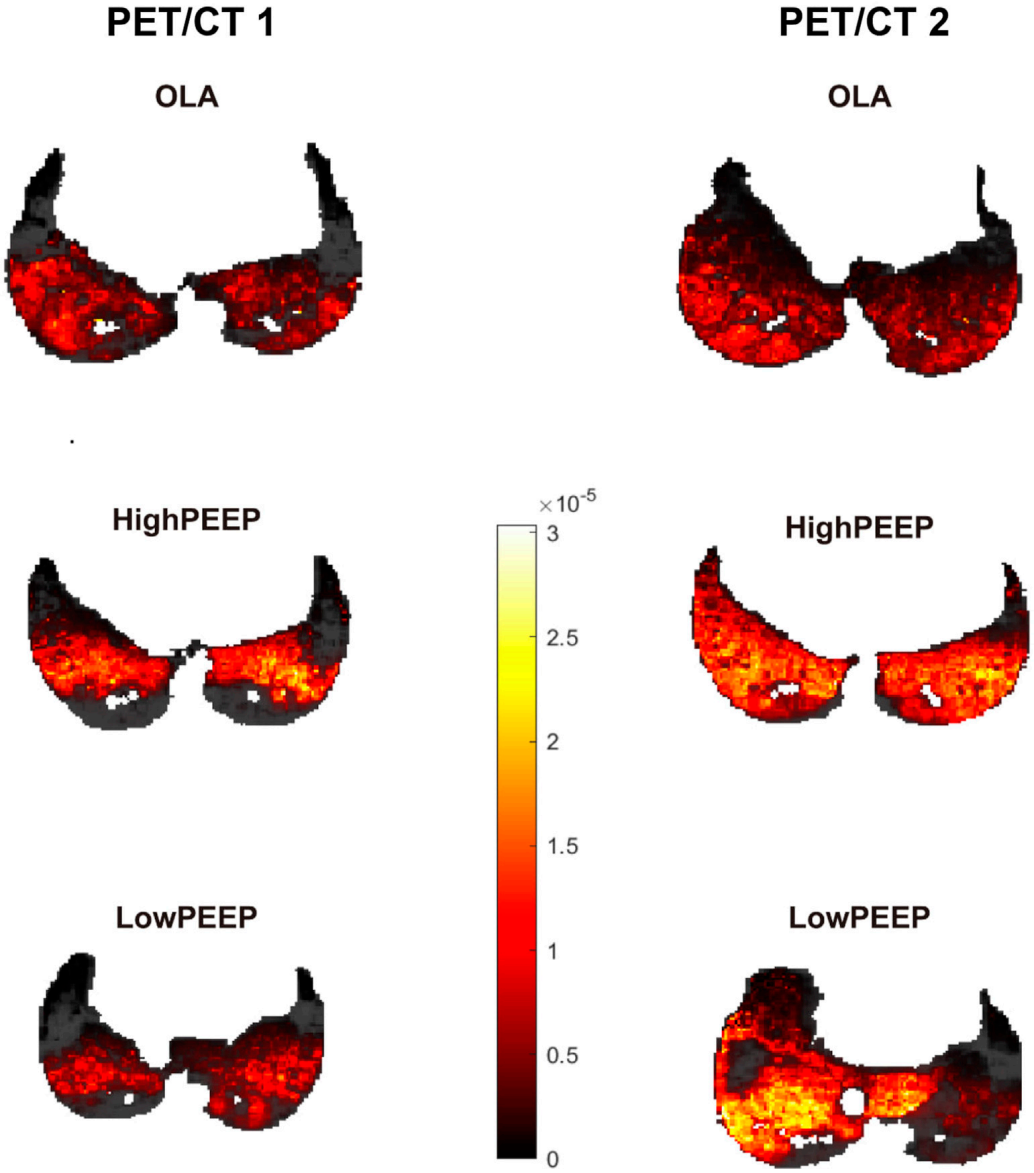
Representative single-slice images of lung perfusion obtained with ^68^Ga-PET scans. According to the perfusion colour bar, higher perfused compartments are indicated by a lighter color while lower perfused compartments are indicated by a darker colour. PET/CT 1, at the start of intervention; PET/CT 2, at the end of intervention.

The pulmonary perfusion of representative animals in each group obtained with ^68^Ga-PET scans is shown in Figure 3. Regional PBF did not differ between groups before (PET/CT 1) and at the end (PET/CT 2) of the intervention time, however, it increased in the LowPEEP group (*P* = 0.012, *P* = 0.012, and *P* = 0.017 for ventral, middle, and dorsal region, respectively) and HighPEEP group (*P* = 0.012 for all) irrespective of lung regions as compared to the start of intervention. In contrast, regional PBF in OLA group was similar (see Supplementary Table S3, supplemental digital content).

### Lung volumes

**FIGURE 4:**
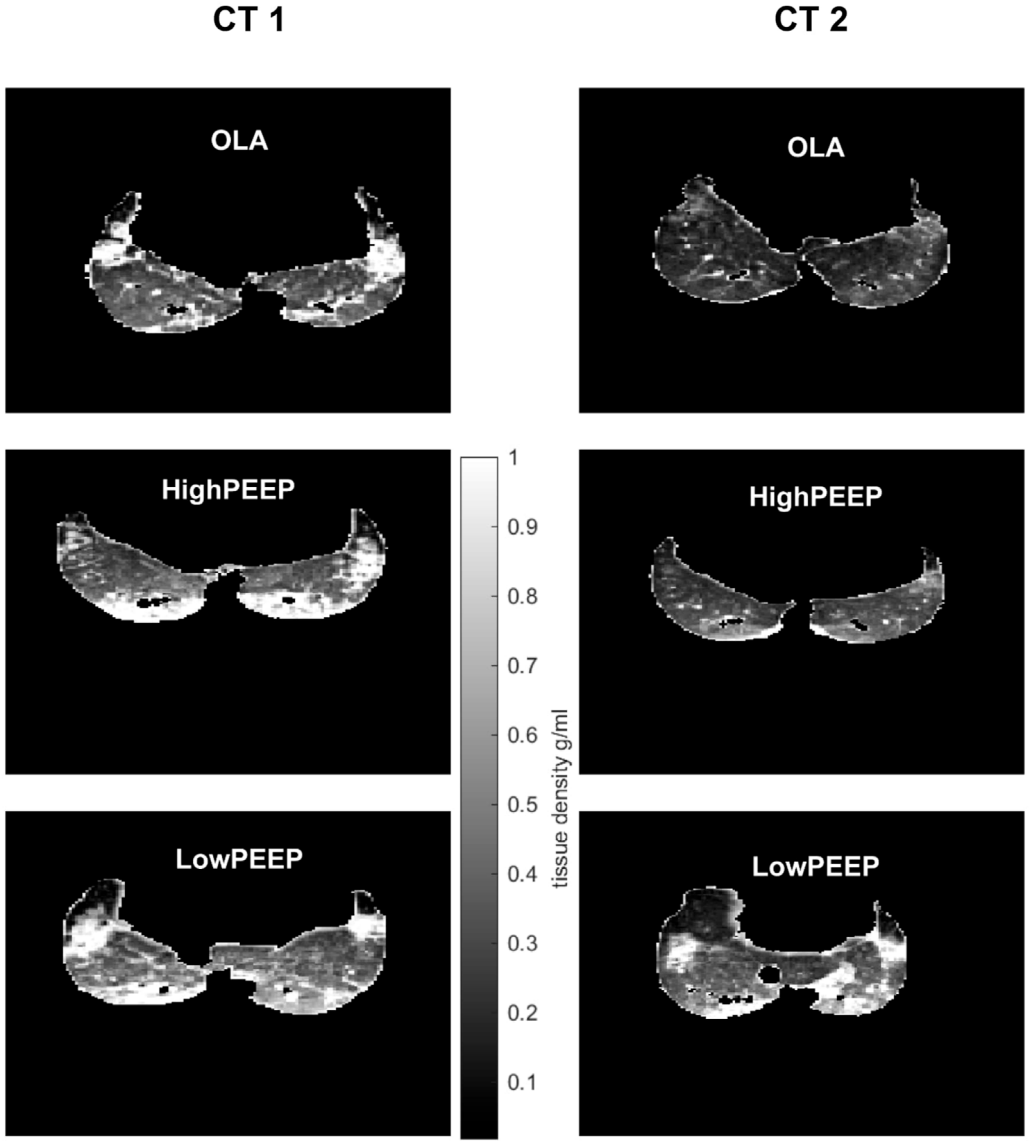
Representative single-slice images of lung density with ACCT. According to the density color bar, hyper-density with lower aerated compartments, i.e., atelectasis, are indicated by white color, while hyper-aerated compartments, i.e., airways, are indicated by black color. CT 1, at the start of intervention; CT 2, at the end of intervention.

Figure 4 shows representative ACCT scans. As compared to OLA and HighPEEP, both end-expiratory lung volume (EELV; 522 ± 98 mL vs. 967 ± 184 mL vs. 911 ± 120 mL, *P* < 0.001 each) and end-inspiratory lung volume (EILV; 857 ± 126 mL vs. 1,252 ± 208 mL vs. 1,222 ± 174 mL, *P <* 0.001 and *P =*0.001, respectively) were lower in the LowPEEP group.

### Correlations analysis

**FIGURE 5:**
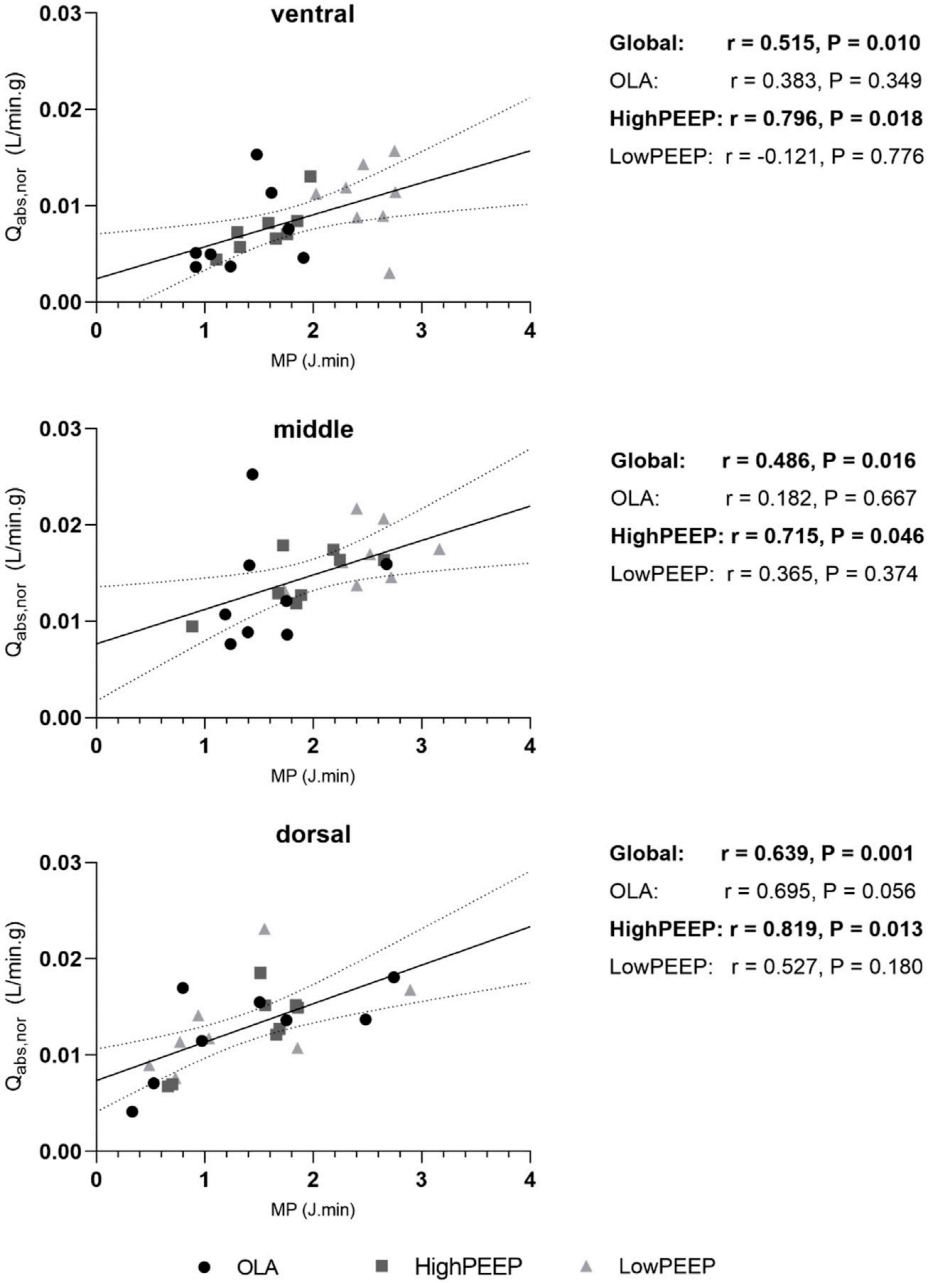
The correlation of regional MP with PBF (Qabs, nor) in three iso-gravitational lung regions. Correlation coefficients (r) are presented on the right panel with *P*-value (Pearson). Interleaved scatters represent paired values of regional MP and PBF. The solid black line represents the best-fit line by linear regression. The dashed lines represent the 95% confidence bands of the best-fit line.

CO was associated with the cumulative volumes of infusion, MPAP, and ventilator parameters. i.e., PEEP and MP (Table 1). However, the association remained significant only with MP after adjustment (0.34 (0.09, 0.59), *P* = 0.020, Table 2). Regional MP was positively associated with PBF irrespective of the different ROIs (0.52 [0.14, 0.76], *P* = 0.010; 0.49 [0.10, 0.74], *P* = 0.016; 0.64 [0.32, 0.83], *P* = 0.001 for ventral, middle, and dorsal ROI, respectively, Figure 5).

**TABLE 1:** The linear regression analysis between CO and the related variables from clinical perspectives.

Item	r [95% CI]	Significance
Infusion (mL)	0.06 (0.02, 0.09)	***P* =0.002**
Norepinephrine (mg)	0.07 (−0.01, 0.16)	*P* = 0.077
Temperature (°C)	−0.25 (−1.62, 1.12)	*P* = 0.707
PEEP (cmH_2_O)	−0.15 (−0.3, −0.01)	***P* = 0.042**
P_plat_ (cmH_2_O)	0.12 (−0.14, 0.39)	*P* = 0.349
P_mean_ (cmH_2_O)	−0.12 (−0.34, 0.1)	*P* = 0.280
MP (J/min)	0.55 (0.36, 0.74)	***P* < 0.001**
MAP (mmHg)	0.04 (−0.05, 0.13)	*P* = 0.345
MPAP (mmHg)	0.18 (0.03, 0.34)	***P* = 0.022**
PCWP (mmHg)	0.08 (−0.17, 0.33)	*P* = 0.509
CVP (cmH_2_O)	−0.02 (−0.25, 0.21)	*P* = 0.873
HR (bpm)	0.02 (−0.01, 0.04)	*P* = 0.195
SV (mL)	0.03 (−0.02, 0.08)	*P* = 0.222

PEEP, positive end-expiratory pressure; MP, mechanical power; MAP, mean arterial pressure; MPAP, mean pulmonary artery pressure; PCWP, pulmonary capillary wedge pressure; CVP, central venous pressure; HR, heart rate; SV, stroke volume. Bold values highlight statistical significance.

**TABLE 2:** Linear multivariable regression analysis of ventilator parameters and CO.

Model	Variable	Crude		Adjusted	
		r [95% CI]	*P* value	r [95% CI]	*P* value
Model1	MP	0.55 (0.37, 0.73)	**<0.001**	0.34 (0.09, 0.59)	**0.020**
Model2	PEEP	−0.15 (−0.29, −0.01)	**0.042**	−0.11 (−0.3, 0.08)	0.284

Model was adjusted for MPAP, HR, infusion, and norepinephrine. Bold values highlight statistical significance.

Subgroup analysis revealed a significant association of MP and CO only in the OLA group (0.79 [0.50, 1.08], *P* = 0.013, Table 3) as well as a significant association of regional MP with PBF only in the HighPEEP group (0.80 [0.37, 0.96], *P* = 0.018; 0.72 [0.03, 0.97], *P* = 0.046; 0.82 [−0.52, 0.99], *P* = 0.013, for ventral, middle, and dorsal ROI, respectively, Figure 5).

**TABLE 3:** Linear univariable and multivariable regression analysis of MP and CO in subgroups.

Subgroup	Crude		Adjusted	
	r [95% CI]	*P*-value	r [95% CI]	*P*-value
OLA	0.74 (0.57, 0.9)	**<0.001**	0.79 (0.5, 1.08)	**0.013**
HighPEEP	0.84 (0.47, 1.21)	**0.004**	0.6 (−0.11, 1.31)	0.195
LowPEEP	0.14 (−0.31, 0.58)	0.570	−0.18 (−0.68, 0.33)	0.536

Correlation was adjusted for MPAP, HR, and infusion. Bold values highlight statistical significance.

## Discussion

In a model of moderate ARDS in pigs, we found that 1) MP was independently associated with CO and positively linearly correlated with regional PBF, nevertheless, a significant association of MP and CO was shown only in the OLA group while a significant association of regional MP with PBF was revealed only in the HighPEEP group; 2) over the intervention time, clinically relevant lung-protective strategies affected lung volumes as well as the distribution of regional MP, but 3) did not result in differences in regional PBF and hemodynamics, except for higher SV during LowPEEP.

This study has several strengths. First, our experimental animal species largely match humans in terms of genetic information, anatomy, and pulmonary physiology (Walters et al., 2017; Woolcock and Macklem, 1971), and allowed highly reproducible measurements of BGA and hemodynamics, including CO, PCWP, and PVR, which are essential determinants of PBF (Russ et al., 2016). Second, ventilation settings using fixed PEEP/F_I_O_2_ tables recommended by ARDS network and OLA, closely resembled the clinical scenarios which are still clinically used (Scharffenberg et al., 2021). Third, we assessed for the first time MP measured at the parenchymal level, which includes multiple factors affecting pulmonary perfusion and thus provides deep insight into the development of VILI. Fourth, we used trapped ^68^Ga-labelled albumine microspheres and PET/CT technique to estimate pulmonary perfusion. The excellent *in vivo* stability, biocompatibility, and high-resolution anatomical information guarantee the precise calculation of PBF (Bailey et al., 2016; Hopkins et al., 2012).

In this study, MP presented a positive but PEEP presented a negative correlation with CO. This result contradicts the MP formula postulated in 2016, which states that MP increases positively with increasing PEEP (Gattinoni et al., 2016), i.e., MP according to the 2016 formula should have been increased at higher PEEP levels. Instead, in the present study, MP as lowest with high PEEP. MP is an emerging respiratory variable that unifies all dynamic and static pressures affecting strain, as well as the rate at which tidal energy is repeated. Despite ample studies approving its superiority to P_plat_ and △P in indicating experimental lung injury and clinical outcomes (Chi et al., 2021; Costa et al., 2021; Cressoni et al., 2016; Scharffenberg et al., 2021; Xie et al., 2022), controversy has arisen mainly regarding the contribution of static elastic components of MP to lung injury (Rocco et al.,2020) and, thus, different calculation methods are discussed in the literature. Considering the sigmoidal relationship between PEEP and elastance, and the fact that the energy associated with PEEP is temporarily stored and dissipated across the exhalation valve before the next inflation cycle begins, MP was calculated using the more sophisticated equation (Equation 1) instead of a simplified one (Huhle et al., 2019; Huhle et al., 2018; Scharffenberg et al., 2021; Zannin et al., 2012). We would like to emphasise that the method of MP calculation has an influence on these results and that future studies are still required in this regard.

After adjustment to MPAP, heart rate (HR), infusion, and norepinephrine, MP instead of PEEP remained significant association with CO. This finding highlights the strength of MP as a comprehensive variable in investigating the invasiveness of mechanical ventilation. The subgroup analysis revealed a significant association only in the OLA group, suggesting that improvements in lung mechanics (manifested as a decrease in MP) in subjects ventilated with OLA are likely to cause hemodynamic instability (manifested as a decrease in CO), which is consistent with our clinical experience. This may be related to the following reasons: First, hemodynamic consequences of a ventilation strategy in injured lungs are primarily influenced by changes secondary to PEEP instead of PEEP *per se* (O’Quin et al., 1985). As a result of improved lung mechanics, more airway pressure is transmitted to the thoracic structures, which more significantly inhibits venous return, thus resulting in lower CO (Grasso et al., 2009). Second, the surfactant depletion model responds well to high airway pressures and RMs, thus ensuring a significant association only in the OLA group. Third, that the cumulatively amounts of crystalloids and colloids did not statistically differ among groups, may be the result of dispersion of data and the relatively small sample size. Of note, this study was not powered for fluid balance and vasopressor needs endpoints, however, the more fragile hemodynamics in the OLA group may have made CO more susceptible to ventilation.

The promising role of total MP in indicating lung injury has been demonstrated, yet increasing evidence suggests that the spatial heterogeneity of lung strain at microscopic levels and regional tissue deformation are the essential determinants of lung injury (Asar et al., 2020; Glenny and Robertson, 2011; Motta-Ribeiro et al., 2019). In this work, we used diffeomorphic transforms and direct differentiation of B-Spline interpolations, a popular technique employed in non-rigid registration of lung CT images, to assess the magnitude of regional MP in three iso-gravitational lung regions. We found that regional MP was positively correlated with PBF irrespective of lung regions. This finding is in line with the well-studied mechanism of $\dot{V}/\dot{Q}$ matching and suggests that higher perfusion would predominate in the regions with greater local MP (Glenny and Robertson, 2011). However, subgroup analyses have revealed a significant association of regional MP with PBF only in the HighPEEP group. Since vasopressor may affect the pumonary vessel pressures, we therefore speculated that higher norepinephrine consumption in the OLA group may alter the pulmonary vascular tone and interfere with the correlations. In addition, we hypothesized that the absence of significant correlation in the LowPEEP group may be related to secondary changes in hydrostatic and transmural pressures. Aria *et al.*, found that changes in hydrostatic and transmural pressure due to changes in vertical lung dimension in the direction of gravity were greater contributors to the lung perfusion redistribution than changes in pulmonary vasculature resistance caused by lung tissue stretch (Arai et al., 2016). Consequently, PBF was redistributed to the region with lower gravity and disturbing ventilation and perfusion ratio (West, 2012).

### Possible clinical implications of the findings

Our findings suggest that during positive pressure MV and in the presence of an increased inflammatory response, MP could serve as an indicator not only for risks of VILI but also for hemodynamics. Clinically relevant lung-protective strategies influence hemodynamics differently. Of note, patients receiving OLA may have fragile hemodynamic stability despite improved lung mechanics. Whereas patients receiving HighPEEP strategy may benefit from reduced transmural pressure due to decreased PBF as a result of improved lung mechanics.

### Limitations

In addition to the common limitations of exploratory analysis, this sub-study has the following limitations. First, the sample size was not estimated specifically for this sub-study. Thus, findings must be interpreted as explorative in nature and some results may indicate differences without statistical significance, e.g., regarding crystalloids and colloids uptake. These non-statistically significant differences should be interpreted with caution. Second, saline lavage impairs lung mechanics by promoting alveolar collapse, yet epithelial damage occurs only when the saline lavage is followed by an injurious ventilation strategy. Induction of lung injury with repetitive saline lavages alone may generate minimal additional stimulus, such as impairment of permeability and limited neutrophil recruitment, and thus direct translation to other ARDS models and clinical scenarios may be precluded (Matute-Bello et al., 2008). Nevertheless, this study allows the investigation of specific important mechanisms. Third, the use of norepinephrine during OLA ventilation may have increased CO and thus artificially influenced the association with MP. Fourth, the timing of lung imaging could not be available for the entire experiment because the nuclear facility is severed for clinical use. Fifth, local PVR and local alveolar partial pressure of oxygen were not available due to technique difficulties, leaving less convincing evidence to explain the mechanisms underlying the correlation between regional MP and PBF. Sixth, this finding suggests that MP, at least in part, leads to lung injury due to increased capillary stress, but we cannot rule out the possibility that CO/PBF is increased simply because of acute lung injury, which is caused by excessive MP.

## Conclusion

In this model of acute lung injury in pigs ventilated with either open lung approach, high, or low PEEP tables recommended by the ARDS network, mechanical power correlated positively with CO and regional PBF, whereby these clinically relevant lung-protective ventilation strategies influenced the associations.

## Trial registration

The protocol of this study was approved by the Landesdirektion Sachsen, Germany (file 25-5131/474/31).

## Summary Statement

In critically ill patients with ARDS, diverse ventilation strategies have been suggested to reverse the life-threatening hypoxemia. However, excessive stress and strain from these strategies can lead to ventilator-induced lung injury, while passive opening and closure of pulmonary capillaries can worsen lung damage due to increased transmural pressures. In addition, the response of the diseased lung to ventilation strategies affects the magnitude of intrathoracic pressure and pulmonary arterial pressure, thereby influencing cardiovascular function. Currently, the optimal ventilation strategy for patients with ARDS remains controversial. Personalized and balanced ventilation strategies based on individual lung-heart interactions are gaining increasing attention. Therefore, indicative parameters that can assess both the risk of lung injury and hemodynamics are urgently needed. This study aims to describe the overall effects of mechanical ventilation on cardiac output and pulmonary blood flow using a single unifying variable, the mechanical power of ventilation, thereby providing clinicians with additional reference points for deciding on the optimal ventilation strategy.

## Data Availability

The raw data supporting the conclusions of this article will be made available by the authors, without undue reservation.

## Glossary

**ACCT**: attenuation-correction computed tomography
**ARDS**: acute respiratory distress syndrome
**BGA**: blood gas analysis
**CO**: cardiac output
**CO**_**Ga**_: cardiac output measured for ^68^Ga-PET/CT
**CT**: computed tomography
**E**_**rs**_: elastance of respiratory system
**E**_**i**_: elastance at the voxel level
**F**_**I**_ **O**_**2**_: inspiratory oxygen fraction
**GLM**: general linear model
**I: E**: inspiratory to expiratory time ratio
**MAP**: mean arterial blood pressure
**MPAP**: mean pulmonary arterial blood pressure
**MP**: mechanical power
**MV**: mechanical ventilation
**OLA**: open lung approach
**PaO**_**2**_: arterial partial pressure of oxygen
**PaO**_**2**_ **/F**_**I**_ **O**_**2**_: Horovitz-index
**PBF**: pulmonary blood flow
**PCWP**: pulmonary capillary wedge pressure
**PEEP**: positive end-expiratory pressure
**PET/CT**: positron-emission tomography/computed tomography
**P**_**peak**_: peak airway pressure
**P**_**plat**_: plateau pressure
**Q**_**abs,ROI**_: absolute lung perfusion at region of interest
**Q**_**abs_nor**_: absolute lung perfusion normalized to lung mass
**R**_**aw**_: resistance of airway
**ROIs**: regions of interest
**RR**: respiratory rate
**SV**: stroke volume
**VILI**: ventilator-induced lung injury
**V**_**T**_: tidal volume
$V_{T,i}$: tidal volume at the voxel level
$\dot{V}/\dot{Q}$: ventilation/perfusion ratio

## References

[B1] Arai T. J. Theilmann R. J. Sa R. C. Villongco M. T. Hopkins S. R. (2016). The effect of lung deformation on the spatial distribution of pulmonary blood flow. J. Physiol. 594 (21), 6333–6347. 10.1113/JP272030 27273807 PMC5088230

[B2] Asar S. Acicbe O. Cukurova Z. Hergunsel G. O. Canan E. Cakar N. (2020). Bedside dynamic calculation of mechanical power: a validation study. J. Crit. CARE 56, 167–170. 10.1016/j.jcrc.2019.12.027 31931417

[B3] Bailey D. L. Eslick E. M. Schembri G. P. Roach P. J. (2016). (68)Ga PET ventilation and perfusion lung imaging-current status and future challenges. SEMINARS Nucl. Med. 46 (5), 428–435. 10.1053/j.semnuclmed.2016.04.007 27553468

[B4] Braune A. (2017). Assessment of the distribution of aeration, perfusion, and inflammation using PET/CT in an animal model of acute lung injury. Med. Fak. Dresd. Disseration Tech. Univ. Available at: https://nbn-resolving.org/urn:nbn:de:bsz:14-qucosa-229255 .

[B5] Chi Y. Zhang Q. Yuan S. Zhao Z. Long Y. He H. (2021). Twenty-four-hour mechanical power variation rate is associated with mortality among critically ill patients with acute respiratory failure: a retrospective cohort study. BMC Pulm. Med. 21 (1), 331. 10.1186/s12890-021-01691-4 34696739 PMC8543779

[B6] Collino F. Rapetti F. Vasques F. Maiolo G. Tonetti T. Romitti F. (2019). Positive end-expiratory pressure and mechanical power. ANESTHESIOLOGY 130 (1), 119–130. 10.1097/ALN.0000000000002458 30277932

[B7] Costa E. L. Slutsky A. Brochard L. J. Brower R. Serpa-Neto A. Cavalcanti A. B. (2021). Ventilatory variables and mechanical power in patients with acute respiratory distress syndrome. Am. J. Respir. Crit. care Med. 204, 303–311. 10.1164/rccm.202009-3467OC 33784486

[B8] Cressoni M. Gotti M. Chiurazzi C. Massari D. Algieri I. Amini M. (2016). Mechanical power and development of ventilator-induced lung injury. ANESTHESIOLOGY 124 (5), 1100–1108. 10.1097/ALN.0000000000001056 26872367

[B9] Dreyfuss D. Saumon G. (1993). Role of tidal volume, FRC, and end-inspiratory volume in the development of pulmonary edema following mechanical ventilation. Am. Rev. Respir. Dis. 148 (5), 1194–1203. 10.1164/ajrccm/148.5.1194 8239153

[B10] Dreyfuss D. Soler P. Basset G. Saumon G. (1988). High inflation pressure pulmonary edema. Respective effects of high airway pressure, high tidal volume, and positive end-expiratory pressure. Am. Rev. Respir. Dis. 137 (5), 1159–1164. 10.1164/ajrccm/137.5.1159 3057957

[B11] Gattinoni L. Tonetti T. Cressoni M. Cadringher P. Herrmann P. Moerer O. (2016). Ventilator-related causes of lung injury: the mechanical power. INTENSIVE CARE Med. 42 (10), 1567–1575. 10.1007/s00134-016-4505-2 27620287

[B12] Glenny R. W. Robertson H. T. (2011). Spatial distribution of ventilation and perfusion: mechanisms and regulation. Compr. Physiol. 1 (1), 375–395. 10.1002/cphy.c100002 23737178

[B13] Grasso S. Stripoli T. Sacchi M. Trerotoli P. Staffieri F. Franchini D. (2009). Inhomogeneity of lung parenchyma during the open lung strategy: a computed tomography scan study. Am. J. Respir. Crit. CARE Med. 180 (5), 415–423. 10.1164/rccm.200901-0156OC 19542479

[B15] Hopkins S. R. Wielputz M. O. Kauczor H. U. (2012). Imaging lung perfusion. J. Appl. Physiol. 113 (2), 328–339. 10.1152/japplphysiol.00320.2012 22604884 PMC3404706

[B16] Hotchkiss J. R. Jr. Blanch L. Murias G. Adams A. B. Olson D. A. Wangensteen O. D. (2000). Effects of decreased respiratory frequency on ventilator-induced lung injury. Am. J. Respir. Crit. CARE Med. 161 (2 Pt 1), 463–468. 10.1164/ajrccm.161.2.9811008 10673186

[B17] Hotchkiss J. R. Jr. Blanch L. Naveira A. Adams A. B. Carter C. Olson D. A. (2001). Relative roles of vascular and airspace pressures in ventilator-induced lung injury. Crit. CARE Med. 29 (8), 1593–1598. 10.1097/00003246-200108000-00016 11505134

[B18] Huhle R. Scharffenberg M. Wittenstein J. J. M. Herzog M. Kiss T. Bluth T. (2019). “Power of ventilation and its relationship with neutrophilic inflammation in a double hit model of acute respiratory distress syndrome,”, D108. American Thoracic Society, A7250. 10.1164/ajrccm-conference.2019.199.1_MeetingAbstracts.A7250

[B19] Huhle R. Serpa Neto A. Schultz M. J. Gama de Abreu M. (2018). Is mechanical power the final word on ventilator-induced lung injury? no. Ann. Transl. Med. 6 (19), 394. 10.21037/atm.2018.09.65 30460268 PMC6212365

[B20] Katira B. H. (2019). Ventilator-induced lung injury: classic and novel concepts. Respir. Care 64 (6), 629–637. 10.4187/respcare.07055 31110032

[B21] Klein S. Staring M. Murphy K. Viergever M. A. Pluim J. P. (2010). elastix: a toolbox for intensity-based medical image registration. IEEE Trans. Med. IMAGING 29 (1), 196–205. 10.1109/TMI.2009.2035616 19923044

[B22] Lopez-Aguilar J. Piacentini E. Villagra A. Murias G. Pascotto S. Saenz-Valiente A. (2006). Contributions of vascular flow and pulmonary capillary pressure to ventilator-induced lung injury. Crit. CARE Med. 34 (4), 1106–1112. 10.1097/01.CCM.0000205757.66971.DA 16484897

[B23] Marini J. J. Hotchkiss J. R. Broccard A. F. (2003). Bench-to-bedside review: microvascular and airspace linkage in ventilator-induced lung injury. Crit. CARE 7 (6), 435–444. 10.1186/cc2392 14624683 PMC374383

[B24] Marini J. J. Rocco P. R. M. Thornton L. T. Crooke P. S. (2024). Stress and strain in mechanically nonuniform alveoli using clinical input variables: a simple conceptual model. Crit. CARE 28 (1), 141. 10.1186/s13054-024-04918-y 38679712 PMC11057067

[B25] Matute-Bello G. Frevert C. W. Martin T. R. (2008). Animal models of acute lung injury. Am. J. physiology. Lung Cell. Mol. physiology 295 (3), L379–L399. 10.1152/ajplung.00010.2008 PMC2536793 18621912

[B26] Moraes L. Silva P. L. Thompson A. Santos C. L. Santos R. S. Fernandes M. V. S. (2018). Impact of different tidal volume levels at low mechanical power on ventilator-induced lung injury in rats. Front. Physiology 9, 318. 10.3389/fphys.2018.00318 PMC5893648 29670537

[B27] Motta-Ribeiro G. Winkler T. Hashimoto S. Vidal Melo M. F. (2019). Spatial heterogeneity of lung strain and aeration and regional inflammation during early lung injury assessed with PET/CT. Acad. Radiol. 26 (3), 313–325. 10.1016/j.acra.2018.02.028 30057194 PMC6612262

[B28] O'Quin R. J. Marini J. J. Culver B. H. Butler J. (1985). Transmission of airway pressure to pleural space during lung edema and chest wall restriction. J. Appl. Physiol. 59 (4), 1171–1177. 10.1152/jappl.1985.59.4.1171 3902775

[B29] Rocco P. R. M. Silva P. L. Samary C. S. Hayat Syed M. K. Marini J. J. (2020). Elastic power but not driving power is the key promoter of ventilator-induced lung injury in experimental acute respiratory distress syndrome. Crit. CARE 24 (1), 284. 10.1186/s13054-020-03011-4 32493362 PMC7271482

[B30] Russ M. Kronfeldt S. Boemke W. Busch T. Francis R. C. Pickerodt P. A. (2016). Lavage-induced surfactant depletion in pigs as a model of the acute respiratory distress syndrome (ARDS). J. Vis. Exp. 115. 10.3791/53610 PMC5091986 27684585

[B31] Scharffenberg M. Wittenstein J. Ran X. Zhang Y. Braune A. Theilen R. (2021). Mechanical power correlates with lung inflammation assessed by positron-emission tomography in experimental acute lung injury in pigs. Front. Physiology 12, 717266. 10.3389/fphys.2021.717266 PMC8645956 34880770

[B32] Shamonin D. P. Bron E. E. Lelieveldt B. P. Smits M. Klein S. Staring M. (2013). Fast parallel image registration on CPU and GPU for diagnostic classification of Alzheimer's disease. Front. Neuroinformatics 7, 50. 10.3389/fninf.2013.00050 PMC3893567 24474917

[B33] Slutsky A. S. Ranieri V. M. (2013). Ventilator-induced lung injury. N. Engl. J. Med. 369 (22), 2126–2136. 10.1056/NEJMra1208707 24283226

[B34] Tustison N. J. Avants B. B. (2013). Explicit B-spline regularization in diffeomorphic image registration. Front. Neuroinformatics 7, 39. 10.3389/fninf.2013.00039 PMC3870320 24409140

[B35] Walters E. M. Wells K. D. Bryda E. C. Schommer S. Prather R. S. (2017). Swine models, genomic tools and services to enhance our understanding of human health and diseases. Lab. Anim. (NY) 46 (4), 167–172. 10.1038/laban.1215 28328880 PMC7091812

[B36] West J. B. (2012). Respiratory physiology: the essentials. 7th ed. Philadelphia, PA: Lippincott Williams and Wilkins, c2005.

[B37] Woolcock A. J. Macklem P. T. (1971). Mechanical factors influencing collateral ventilation in human, dog, and pig lungs. J. Appl. PHYSIOLOGY 30 (1), 99–115. 10.1152/jappl.1971.30.1.99 5538799

[B38] Xie Y. Zheng H. Mou Z. Wang Y. Li X. (2022). High expression of CXCL10/CXCR3 in ventilator-induced lung injury caused by high mechanical power. Biomed. Res. Int. 2022, 6803154. 10.1155/2022/6803154 35036436 PMC8759875

[B39] Zannin E. Dellaca R. L. Kostic P. Pompilio P. P. Larsson A. Pedotti A. (2012). Optimizing positive end-expiratory pressure by oscillatory mechanics minimizes tidal recruitment and distension: an experimental study in a lavage model of lung injury. Crit. CARE 16 (6), R217. 10.1186/cc11858 23134702 PMC3672594

