# Supplementary material for: Mechanical power is associated with cardiac output and pulmonary blood flow in an experimental acute respiratory distress syndrome in pigs
Source: Front Physiol. 2024 Oct 15;15:1462954. doi: 10.3389/fphys.2024.1462954 (PMC11519626; doi:10.3389/fphys.2024.1462954)
Supplement: Supplementary file 1 [file DataSheet1.pdf]

Mechanical power is associated with cardiac output and pulmonary blood flow in an experimental acute respiratory distress syndrome in pigs

Yingying Zhang, Jakob Wittenstein, Anja Braune, Raphael Theilen, Lorenzo Maiello, Giulia Benzi, Thomas Bluth, Thomas Kiss, Xi Ran, Thea Koch, Patricia R. M. Rocco, Marcus J. Schultz, Jörg Kotzerke, Marcelo Gama de Abreu, Robert Huhle, Martin Scharffenberg

Supplemental digital content

Methods

PEEP-Titration in the OLA group:

RMs were initiated using APRV with driving pressure of 15 cmH_2O, PEEP of 5 cmH_2O, RR of 10/min, I:E of 1:1, and F_IO_2 of 1.0. PEEP was increased in steps of 5 cmH_2O every 10 cycles until PEEP of 25 cmH_2O and maintained at the last step for 15 cycles. Ventilation mode was switched to IPPV with V_T of 6 ml/kg, RR of 15/min, I:E of 1:1, and F_IO_2 of 1.0. The initial PEEP of 24 cmH_2O was reduced in steps of 2 cmH_2O every 30 s until the highest Crs was detectable. Crs was noted at the end of each step. The “best-PEEP” was considerated as the level of PEEP presenting the highest compliance and plus 2 cmH_2O. Afterwards, another RM was performed as previously described. Then, previous MV settings were resumed but “best-PEEP” kept (supplement figure 1).

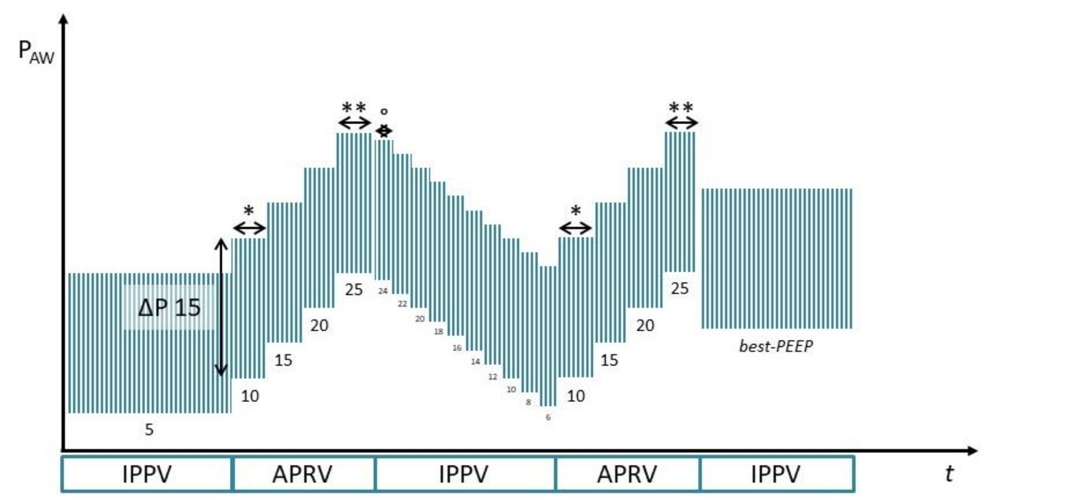

Supplemental figure: RM and PEEP titration; * 10 cycles; ** 15 cycles; ° 30 s

Supplement figure 1: RM and PEEP titration. IPPV: intermittent positive pressure ventilation; APRV: airway pressure release ventilation; PEEP: positive end-expiratory pressure; △P: driving pressure; PAW: airway pressure.*: maintain for 10 breath cycles; **: maintain for 15 breath cycles; °: maintain for 30 seconds.

PEEP/F_IO_2-Setting in High/LowPEEP groups:

After randomization to the HighPEEP or the LowPEEP group, 20-30 min of sham ventilation was performed to compensate for the titration duration in the OLA group. The lowest possible PEEP/F_IO_2 combination, which maintained PaO_2 of 55-80 mmHg, was set within this sham time. Arterial blood samples were checked hourly for the adjustments of PEEP/F_IO_2 combination according to ARDSnet-Tables during the whole interventional time (supplement table 1).

Supplement table 1: PEEP/F_IO_2 combinations

HighPEEP Table										
F_IO_2	0.3	0.3	0.4	0.4	0.5	0.5	0.5-0.8	0.8	0.9	1.0
PEEP	12	14	14	16	16	18	20	22	22	22-24
**LowPEEP Table**										
F_IO_2	0.3	0.4	0.4	0.5	0.5	0.6	0.7	0.8	0.9	1.0
PEEP	5	5	8	8	10	10	10-14	14	14-18	18-24

PEEP: Positive end-expiratory pressure; F_IO_2: Fraction of inspired oxygen

Results

General Aspects

Twenty-four animals were included in the final analysis. There were no differences between OLA, HighPEEP and LowPEEP groups regarding body weight (39.1 ± 5.6 kg vs. 41.3 ± 6.1 kg vs. 46.0 ± 5.5 kg, P =0.067), number of lavages to meet the aimed criteria (9 ± 2 vs. 10 ± 3 vs. 9 ± 2, P =0.430), and duration of anaesthesia (34.2 ± 0.7 h vs. 34.0 ± 0.4 h vs. 33.7 ± 0.3 h, P =0.374). In total, animals in the three groups received similar cumulative volumes of crystalloids (191 [12] ml/kg vs. 180 [11] ml/kg vs. 174 [4] ml/kg, P =0.059) and colloids (13 [47] ml/kg vs. 6 [20] ml/kg vs. 0 [0] ml/kg, P =0.065). Groups also did not differ regarding urine output (1.8 ± 0.4 ml/kg/h vs. 2.0 ± 0.9 ml/kg/h vs. 2.3 ± 1.0 ml/kg/h, P =0.537). During the intervention time, the mean body temperature did not differ between groups (38.8 ± 0.2 °C vs. 38.9 ± 0.2 °C vs. 38.8 ± 0.2 °C, P =0.796). However, animals received less intervention time in the OLA group than in the HighPEEP group (22.5 ± 0.2 h vs. 23.1 ± 0.4 h, P =0.003), and consumed cumulatively higher doses of norepinephrine compared to the LowPEEP group (0 [0] mg/kg vs. 0.03 [0.35] mg/kg, P =0.038), respectively.

Mean Airway Pressure

Supplement table 2: Mean airway pressure [cmH_2O] during the intervention time.

Group	T_{0h}	T_{3h}	T_{6h}	T_{9h}	T_{12h}	T_{15h}	T_{18h}	Group *P=*	Time *P=*	G*T *P=*
OLA	18±3	19±2	19±2	18±2	18±2	19±2	19±2	**<0.001**	**0.029**	0.117
High PEEP	19±1	19±1	18±1	18±1	18±1	18±1	18±1			
Low PEEP	13±1	14±1	14±1	14±1	14±1	14±1	14±1			

G*T Group time effect. OLA and HighPEEP vs. LowPEEP P<0.001 each; OLA vs. HighPEEP P>0.999

Gas Exchange and Shunt

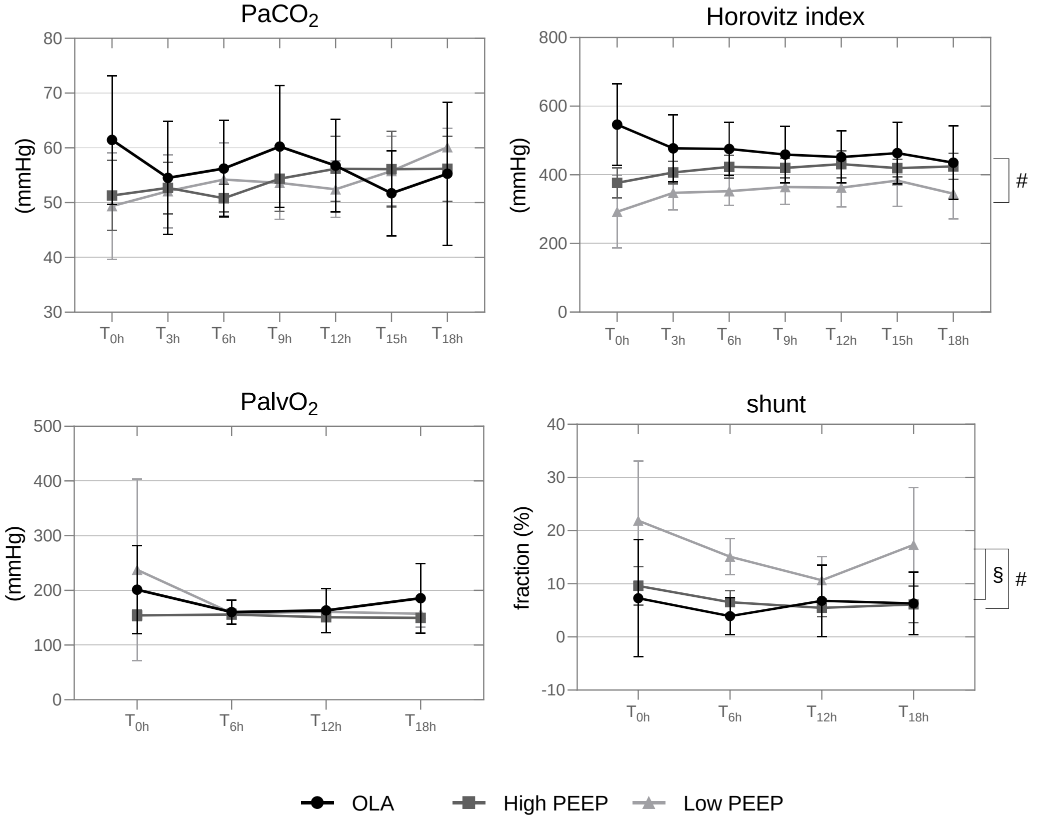

Supplement figure 2: Time course of gas exchange variables throughout the intervention time. Interleaved symbols are presented in mean with SD. T_{0h} is the start time of intervention, T_{3h-18h} are repeated measurements performed every three hours from T_0 onwards. $PaCO_2$: Arterial carbon dioxide partial pressure, $PalvO_2$: Alveolar partial pressure of oxygen, Horovitz index: the ratio between PaO_2 and F_iO_2. GLM was adopted for group comparison over intervention time (T_{0h-18h}). The correction according to Šidák was used. #: comparison between OLA and LowPEEP. §: comparison between LowPEEP and HighPEEP groups.

Hemodynamics

Supplement table 3 shows hemodynamic variables. Not only cardiac output, but also cardiac index showed a significant positive correlation with MP (r=0.688; 95%CI 0.393-0.854; P<0.001).

Supplement table 3: Haemodynamics measured over time

Group	T_{0h}	T_{3h}	T_{6h}	T_{9h}	T_{12h}	T_{15h}	T_{18h}	Group	Time	Group*Time
HR [bpm]										
OLA	120 ± 34	115 ± 19	115 ± 21	129 ± 46	121 ± 39	122 ± 36	119 ± 36	*P* =0.206	*P* =0.153	*P* =0.246
HighPEEP	106 ± 21	109 ± 24	109 ± 15	108 ± 25	107 ± 25	106 ± 26	104 ± 26			
LowPEEP	117 ± 17	106 ± 14	101 ± 10	96 ± 7	96 ± 8	92 ± 7	103 ± 14			
CO [l/min]										
OLA	4.5 ± 1.4	4.3 ± 1.7	4.3 ± 1.7	4.7 ± 1.3	5.0 ± 1.5	4.8 ± 1.2	5.1 ± 1.4	*P* =0.065	**P =0.005**	*P* =0.603
HighPEEP	4.6 ± 1.1	4.7 ± 1.5	5.0 ± 1.4	5.2 ± 1.4	5.3 ± 1.4	5.5 ± 1.6	5.8 ± 1.6			
LowPEEP	6.2 ± 1.3	6.2 ± 1.3	6.2 ± 1.3	6.2 ± 1.0	5.9 ± 1.2	5.9 ± 1.1	6.9 ± 1.2			
MAP [mmHg]										
OLA	76 ± 9	71 ± 9	68 ± 8	69 ± 6	69 ± 6	68 ± 7	70 ± 7	*P* =0.072	***P* < 0.001**	*P* =0.094
HighPEEP	75 ± 7	70 ± 7	68 ± 6	66 ± 5	68 ± 9	70 ± 7	68 ± 7			
LowPEEP	82 ± 9	76 ± 7	74 ± 7	73 ± 6	73 ± 5	77 ± 6	78 ± 8			

Continued on next page.

Table 3: (continued)

Group	T_{0h}	T_{3h}	T_{6h}	T_{9h}	T_{12h}	T_{15h}	T_{18h}	Group	Time	Group*Time
MPAP [mmHg]										
OLA	22 ± 5	23 ± 7	23 ± 6	23 ± 5	22 ± 6	20 ± 4	22 ± 4	*P* =0.204	***P* =0.01**	***P* =0.031**
HighPEEP	24 ± 2	22 ± 2	20 ± 2	20 ± 2	20 ± 2	19 ± 3	20 ± 2			
LowPEEP	26 ± 4	24 ± 3	24 ± 3	23 ± 3	23 ± 3	22 ± 3	25 ± 6			
PCWP [mmHg]										
OLA	9 ± 2	9 ± 3	10 ± 3	10 ± 4	11 ± 3	11 ± 3	12 ± 3	*P* =0.216	***P* < 0.001**	*P* =0.490
HighPEEP	8 ± 3	9 ± 3	9 ± 2	10 ± 3	9 ± 3	9 ± 3	10 ± 2			
LowPEEP	7 ± 2	8 ± 2	8 ± 2	8 ± 2	9 ± 2	9 ± 3	8 ± 2			
CVP [cmH_2O]										
OLA	7 ± 2	8 ± 3	8 ± 3	9 ± 5	9 ± 4	8 ± 2	10 ± 3	*P* =0.059	***P* < 0.001**	*P* =0.464
HighPEEP	6 ± 3	5 ± 2	7 ± 3	7 ± 3	7 ± 2	7 ± 2	7 ± 3			
LowPEEP	5 ± 2	4 ± 1	5 ± 2	5 ± 2	6 ± 3	7 ± 3	6 ± 2			

Continued on next page.

Table 3: (continued)

Group	T_{0h}	T_{3h}	T_{6h}	T_{9h}	T_{12h}	T_{15h}	T_{18h}	Group	Time	Group*Time
*PVR [mmHg * min / l]*										
OLA	242 ± 142	271 ± 107	224 ± 71	215 ± 51	180 ± 43	159 ± 34	159 ± 43	*P* =0.936	***P* =0.006**	***P* =0.034**
HighPEEP	300 ± 134	257 ± 104	191 ± 60	179 ± 85	178 ± 71	158 ± 57	155 ± 50			
LowPEEP	246 ± 69	222 ± 60	206 ± 75	207 ± 65	190 ± 53	185 ± 34	192 ± 62			
CI [l/min/m^2]#										
OLA	4.8 ± 1.2	4.6 ± 1.5	5.3 ± 1.7	5.2 ± 1.1	5.4 ± 1.3	5.3 ± 1.1	5.6 ± 1.3	*P* =0.159	***P* =0.003**	P =0.318
HighPEEP	4.7 ± 0.8	4.8 ± 1.2	5.2 ± 1.2	5.3 ± 1.2	5.5 ± 1.2	5.7 ± 1.5	6.0 ± 1.4			
LowPEEP	6.1 ± 1.0	6.1 ± 0.9	6.3 ± 1.0	6.0 ± 0.8	5.8 ± 1.1	5.8 ± 1.1	6.8 ± 1.1			
SV *[ml]*										
OLA	37 ± 7	39 ± 11	43 ± 9	41 ± 9	45 ± 9	44 ± 11	47 ± 9	***P* =0.002**	***P* < 0.001**	*P* =0.312
HighPEEP	41 ± 11	42 ± 11	45 ± 12	46 ± 13	49 ± 12	50 ± 13	53 ± 13	Post hoc:		
LowPEEP	54 ± 8	58 ± 11	63 ± 14	64 ± 11	61 ± 9	61 ± 10	67 ± 11	#: ***P* =0.002**		
								§: ***P* =0.018**		

CO: cardiac output; CI: cardiac index; MAP: mean arterial pressure; PCWP: pulmonary capillary wedge pressure; CVP: central venous pressure; PVR: pulmonary vascular resistance; SV: stroke volume. T_{0h} is the start time of intervention, T_{3h} to T_{18h} are repeated measurements performed every three hours from T_0 onwards. #: comparison between OLA and LowPEEP. §: comparison between LowPEEP and HighPEEP groups. Bold font highlights the significant difference. GLM was adopted for group comparison over intervention time ($T_{0h\text{-}18h}$). The correction according to Šidák was used.

CI= CO/Body surface area, with body surface area=0,0798*Bodyweight^(2/3)

Lung Perfusion

The supplemental figure 3, with panels A to F, shows the comparisons of regional PBF (Qabs_nor) between OLA, HighPEEP, and LowPEEP groups, and between the start (day1) and the end of intervention time (day2).

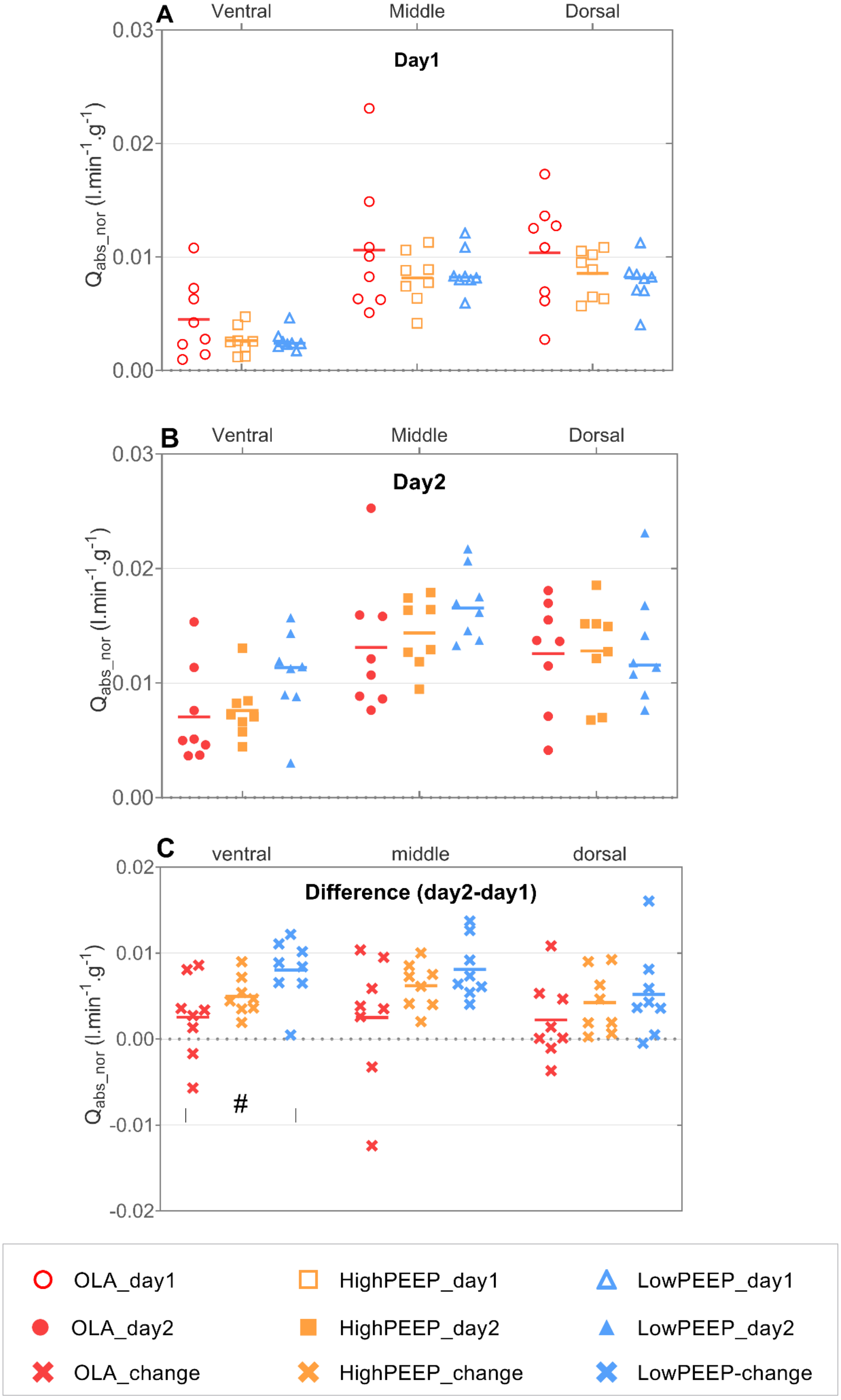
A
Ventral
Middle
Dorsal
Day1
Q_{abs_nor} (l.min^{-1}.g^{-1})
0.03
0.02
0.01
0.00
B
Ventral
Middle
Dorsal
Day2
Q_{abs_nor} (l.min^{-1}.g^{-1})
0.03
0.02
0.01
0.00
C
ventral
middle
dorsal
Difference (day2-day1)
Q_{abs_nor} (l.min^{-1}.g^{-1})
0.02
0.01
0.00
-0.01
-0.02
#
OLA_day1
HighPEEP_day1
LowPEEP_day1
OLA_day2
HighPEEP_day2
LowPEEP_day2
OLA_change
HighPEEP_change
LowPEEP-change

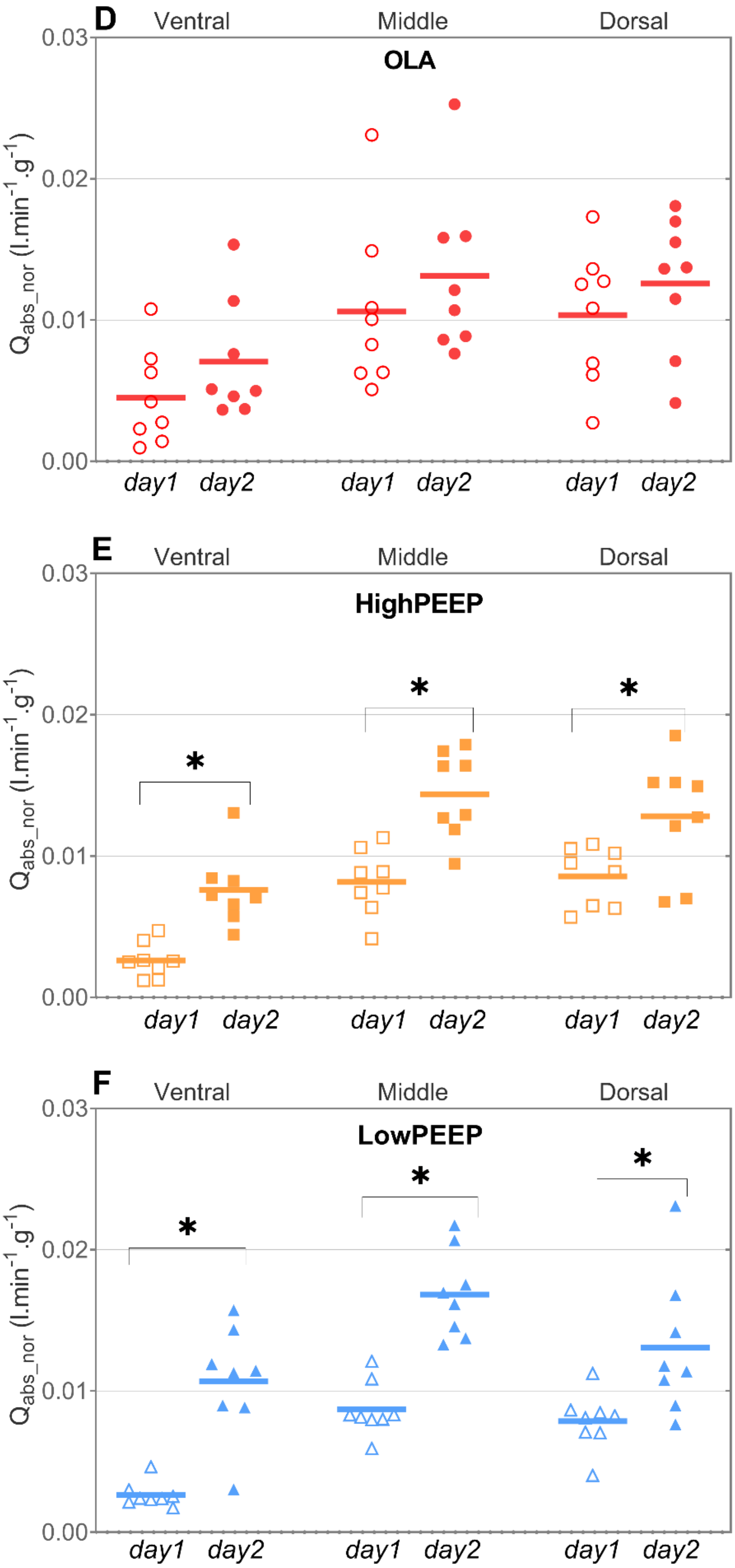

Supplement figure 3: Comparisons of regional PBF (Qabs_nor) between OLA, HighPEEP, and LowPEEP groups, and between the start (day1) and the end of intervention time (day2). Regional PBF were presented according to time (A and B) and ventilation strategies (D, E, and F). C is the difference

in regional PBF between day1 and day2. Interleaved scatters represent the individual values with red dots standing for OLA, yellow squares standing for HighPEEP, and blue triangles standing for LowPEEP. Open and filled icons present values measured before the start and at the end of intervention, respectively. Cross icons present the difference in Q_{abs_nor}. The horizontal lines represent median values. Wilcoxon and Kruskal-Wallis were used for comparisons as appropriate. *: significant difference between day1 and day2. #: significant difference between OLA and LowPEEP group. The correction according to Bonferroni was used.
